# The Mechanism of Phillyrin from the Leaves of* Forsythia suspensa* for Improving Insulin Resistance

**DOI:** 10.1155/2019/3176483

**Published:** 2019-07-02

**Authors:** Xiaoqing Xu, Fatma S. A. Saadeldeen, Lanting Xu, Yingying Zhao, Jinfeng Wei, Hui-Min David Wang, Zhenhua Liu, Wenyi Kang

**Affiliations:** ^1^National R & D Center for Edible Fungus Processing Technology, Henan University, Kaifeng 475004, China; ^2^Joint International Research Laboratory of Food & Medicine Resource Function, Henan Province, Kaifeng 475004, China; ^3^Medicinal and Aromatic Plant Research Department, Horticulture Research Institute, Agricultural Research Centre, Cairo 12611, Egypt; ^4^Graduate Institute of Biomedical Engineering, National Chung Hsing University, Taichung City 402, Taiwan

## Abstract

Three lignans, phillyrin, forsythia ester A, and rosin-*β*-D-furan glucose, were isolated from* Forsythia suspensa* which is a famous Traditional Chinese Medicine used for clearing heat and detoxifying, reducing swelling and dispersing knot, and dispersing wind heat. In this study, the effects of phillyrin, forsythia ester A, and rosin-*β*-D-furan glucose on insulin resistance of 3T3-L1 adipocytes were investigated by the method of glucose oxidase-peroxidase (GOD-POD) and the mechanism was assayed by the method of western blot. The results indicated that phillyrin, forsythia ester A, and rosin-*β*-D-furan glucose could improve the glucose uptake in 3T3-L1 adipocytes under insulin resistance (IR). Among them, phillyrin showed significant activity in increasing glucose consumption at the concentrations of 100 *μ*M and 200 *μ*M (*P* < 0.001). The mechanism of improving insulin resistance may be that phillyrin could raise the protein phosphorylation of IRS-1 and Akt and the expression levels of GLUT4 protein.

## 1. Introduction

Type 2 Diabetes (T2DM) is characterized by insulin resistance (IR) and destruction of *β* cells in pancreas [1–3]. IR refers to the reduction of insulin in regulating glucose homeostasis in its main target tissues (such as skeletal muscle, fat, and liver) [4, 5]. It is not only an important pathophysiological mechanism of the onset of T2DM, but also the common basis of atherosclerosis, metabolic syndrome, and other diseases [6, 7]. Therefore, how to improve the sensitivity of insulin has become a key goal in the treatment of T2DM.

Insulin as a key drug for the treatment of T2DM binds to the corresponding receptor of the target cell and causes the phosphorylation of tyrosine residue and then activates *β* subunits of tyrosine protein kinase. Then, tyrosine protein kinase is activated to make the target cell substrates such as “IRS” and “SHC” phosphorylate tyrosine residues. Also, tyrosine protein kinase plays an important role in insulin receptor signaling [8, 9]. These events finally activate insulin signaling pathway, including insulin receptor substrate 1-Rat sarcoma-mitogen activation protein kinase (IRS-RaS-MAPK) and insulin receptor substrate 1-phosphatidylinositol-3 kinase-protein kinase B (IRS-1/PI3K/Akt) [10]. The former mainly regulates cell growth and apoptosis. The latter participates in metabolic effects, like glucose transport and utilization, glycogen synthesis, and the like, which plays a very important role in the development of IR. And it is a very important signaling pathway in the study of T2DM. In the insulin signaling pathway, insulin binds to InsR, which is phosphorylated by its own tyrosinase to activate IRS-1, and the phosphorylated IRS binds the regulatory subunit P85 of phosphatidylinositol 3-kinase (PI-3K) [11, 12], further activating its catalytic subunit P110 [13, 14]. The IRS-1 protein is the main substrate of insulin receptor, and IRS-1 binds to the Tyr sequence, which is phosphorylated by the insulin receptor molecule through its SH2 region, thereby triggering further phosphorylation [15, 16]. Activated PI3K phosphorylates serine-threonine kinase (Akt) [17, 18], and phosphorylated Akt stimulates GLUT4 in vesicles transferring from cytoplasm to cell membrane, thus promoting the uptake and utilization of glucose to various tissues [9, 19, 20]. Therefore, it is important to reduce IR and promote glucose metabolism for T2DM.

*Forsythia suspensa* is a famous Traditional Chinese Medicine with the functions of clearing heat and detoxifying, reducing swelling and dispersing knot, and dispersing wind heat [21, 22].* F. suspensa* also has many pharmacological activities, such as antioxidation, anti-infection, antibacterial, antiendotoxin, heart protection, anti-inflammatory, liver protection, antivirus, and antifatigue [23–25]. Our previous researches showed that the ethyl acetate extract of* F. suspensa* and its chemical components had the effect of lowering blood lipid [26]. The main active ingredients of* F. suspensa* were phillyrin, oleanolic acid, forsythia lipid, etc. [21]. However, there have been no reports on the mechanism of the active ingredients of* F. suspensa* to improve IR. Therefore, mouse embryonic preadipocytes 3T3-L1 adipocytes were used to investigate the activities and their mechanisms of phillyrin, forsythia ester A (FEA), and rosin-*β*-D-furan glucose (RFG) from the leaves of* F. suspensa* for improving insulin resistance.

## 2. Materials and Methods

### 2.1. Cell Culture

Mouse 3T3-L1 preadipocyte cells (TCSC Culture Collection) were grown in high glucose (4.5 mM) DMEM (glutamine, 10 *μ*g/mL penicillin streptomycin, 10 U/mL, pyruvic acid, and ammonium) with 10% FBS at 37°C in 5% CO_2_ atmosphere and cell fusion rate of 70% ~ 80%, extending the ratio of 1:3 [27].

### 2.2. Cell Viability Assay

3T3-L1 preadipocytes were seeded into 96-well plates (cell density was 5×10^3^ cells per well) and cultured for 24 h. Cells were treated with different concentrations of phillyrin, FEA, and RFG (concentration of 400, 200, 100, 50, 25, 12.5, and 6.25 *μ*M ) for 48 h; after that, MTT (3-(4,5-dimethylthiazol-2-yl)-2,5-diphenyltetrazolium bromide) 0.5 mg/mL was added; then cells were maintained in an incubator at 37°C for 4 h. After discarding the supernatant, the insoluble formazan crystals were dissolved by 100 *μ*L dimethyl sulfoxide (DMSO) to each well, and the plates were shaken for 10 min. Absorbance was measured by a microplate reader (Thermo Fisher, Finland) at 490 nm. Control cells were arbitrarily assigned 100% viability.

### 2.3. Determination of Insulin Resistance to Adipocyte Glucose Uptake

The cells were cultured in 96-well plates (cell density was 2×10^4^), and the medium was changed 24 hours later. At this time, cell contact inhibition was observed. After the inhibition of cell contact, medium was changed to 10% FBS DMEM supplemented with 0.5 mM 3-isobutyl-1-methylxanthine (IBMX), 1 mM dexamethasone (Dex), and 10 *μ*g/mL insulin. 3 days later, the medium was replaced with DMEM supplemented with 15% FBS and insulin (10 mg/L) and cultured for 2 days, and then the medium was changed to normal culture medium and cultured for another for 4 days. After inducing differentiation into mature adipocytes, 1 *μ*M Dex was used for modeling for 72 h. After that, 10 mol/L sodium orthovanadate was used as positive control drug and different concentrations of phillyrin, FEA, and RFG were cultured for 48 h. The method of glucose oxidase-peroxidase (GOD-POD) was used to determine the level of glucose in the cell supernatant by a glucose assay kit (Shanghai Rongsheng Biotech Co.) according to the manufacturer's instructions.

### 2.4. Western Blot Analysis

Cells harvested with different concentrations of phillyrin for 48 h were collected and lysed on ice for 30 min in a mixture containing Radio-Immunoprecipitation Assay (RIPA), phenylmethane sulfonyl fluoride (PMSF), and phosphatase inhibitors. The lysate was centrifuged at 12000 rpm for 10 min at 4°C. Protein concentration was measured by a BCA protein assay kit (Solarbio Science & Technology Co., Ltd.). After quantitative protein sample, it was added to 5 x loading buffer, under 100°C high temperature degeneration. Protein samples at the same amount (50/70 *μ*g) were separated on 10% SDS-polyacrylamide gel. Proteins were transferred onto polyvinylidene fluoride (PVDF) membranes (0.2 *μ*M, EMD Millipore, Billerica, MA, USA), which were blocked in 5% nonfat dry milk for 2 h. The membrane added to specific primary antibodies was diluted in TBST overnight at 4°C. Then, it was incubated with horseradish peroxidase conjugated secondary antibody for 1 h. After wash, the protein of interest was identified by an ECL Plus Ultrasensitive Liquid.

### 2.5. Statistical Analysis

The experimental results were expressed by arithmetic means ± standard deviation (SD), and the numerical statistics were analyzed by SPSS 19.0 software one-way analysis of variance.* P*≤0.05 was considered to be statistically significant. All bar images were analyzed by GraphPad Prism 6.0 software.

## 3. Results

### 3.1. Effects of Phillyrin, FEA, and RFG on 3T3-L1 Cell Viability

**Figure 1:**
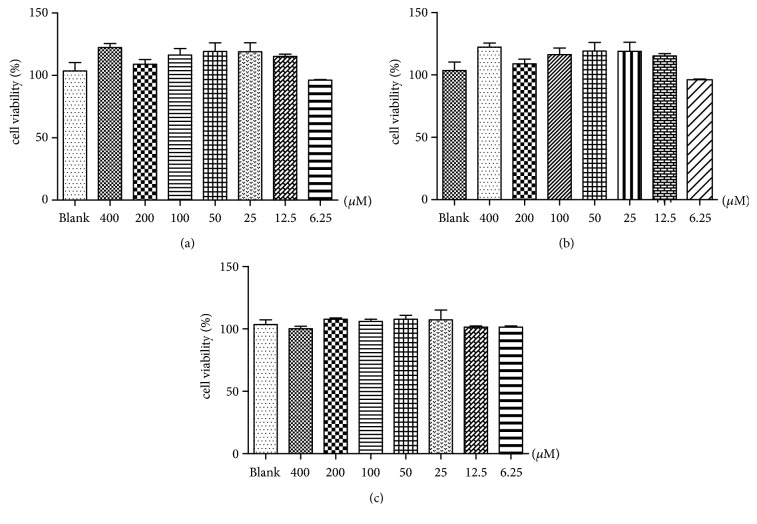
(a) Effect of phillyrin on the viability of 3T3-L1 adipocytes. (b) Effect of FEA on the viability of 3T3-L1 adipocytes. (c) Effect of RFG on the viability of 3T3-L1 adipocytes. Data are presented as means ± SD,* n* = 6.

The cytotoxic effects of phillyrin, FEA, and RFG on cell viability were evaluated by MTT assay. Phillyrin, FEA, and RFG all showed no cytotoxicity at concentrations less than 400 *μ*M (Figure 1).

### 3.2. Effects of Phillyrin, FEA, and RFG on IR in Adipocyte Glucose Depletion

**Figure 2:**
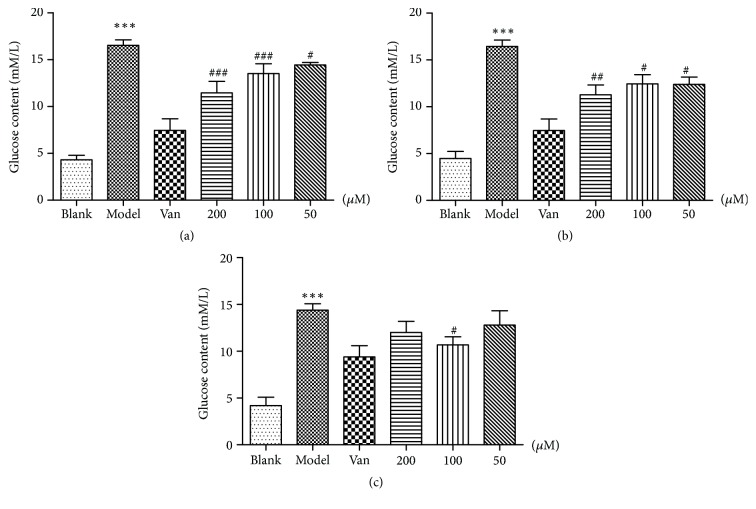
(a) Effects of phillyrin on glucose consumption in IR adipocytes, (b) effects of FEA on glucose consumption in IR adipocytes, and (c) effects of RFG on glucose consumption in IR adipocytes. Data are presented as means ± SD,* n *= 6. ^*∗∗∗*^*P* < 0.001 versus blank group;^ ###^*P *< 0.001, ^##^*P* < 0.01, and ^#^*P* < 0.5 versus model group.

The glucose content of the supernatant was measured after 48 h of exposure to phillyrin, FEA, and RFG at different concentrations with the glucose oxidase kit. The model exhibited a significant difference (*P*<0.001) compared with the control group. Sodium orthovanadate (Van) could significantly promote IR in adipocytes glucose consumption (*P*<0.001) compared with the model group. Phillyrin could significantly promote IR in adipocytes glucose consumption at the concentrations of 200 and 100 *μ*M (*P* < 0.001) (Figure 2(a)). FEA could promote the glucose uptake of IR 3T3-L1 adipocytes at the concentrations of 200, 100 (*P*<0.01), and 50 *μ*M (*P*<0.5) (Figure 2(b)). The glucose uptake of IR 3T3-L1 adipocytes was promoted by RFG at the concentrations of 100 *μ*M (*P*<0.5), but the effect was not as obvious as phillyrin (Figure 2(c)).

### 3.3. Effects of Phillyrin on the Expression of IRS-1 and Akt Phosphorylation Proteins

**Figure 3:**
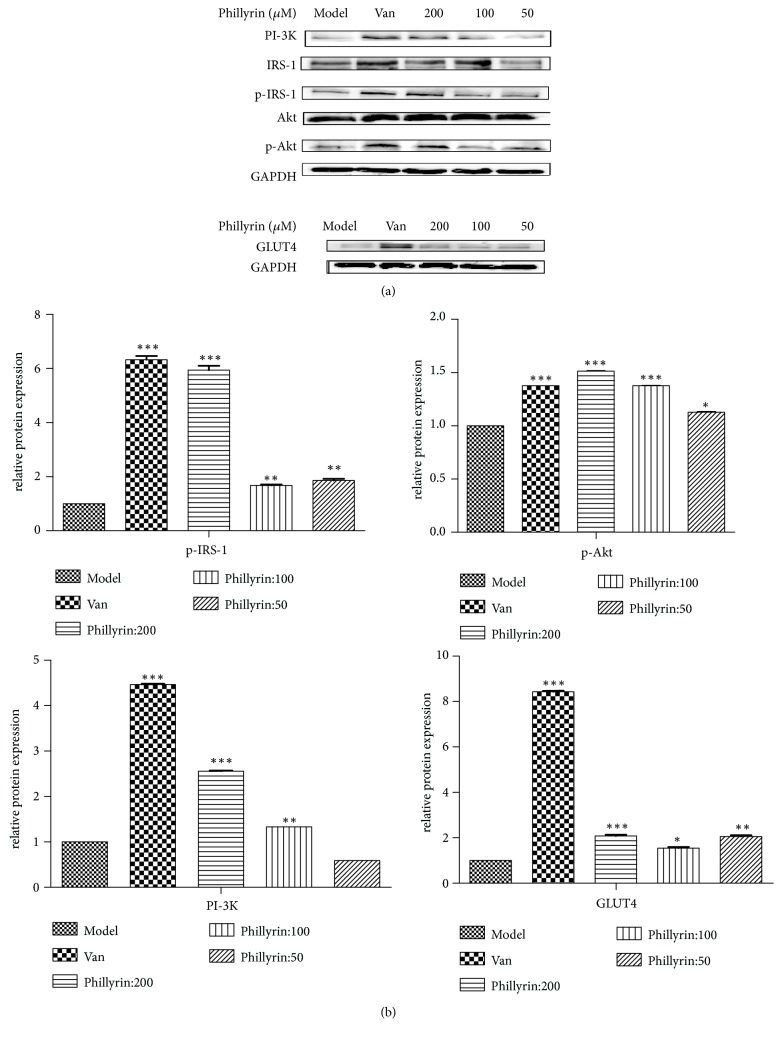
Western blot analysis of protein expression. (a) Effects of phillyrin on protein expression levels of PI-3K, IRS-1, p-IRS-1, Akt, p-Akt, GAPDH, and GLUT4 in IR 3T3-L1 adipocytes. (b) Gray value analysis of corresponding protein. Data are presented as means ± SD,* n* = 6. The experiments were repeated three times. ^*∗∗∗*^*P* < 0.001, ^*∗∗*^*P* < 0.1, and ^*∗*^*P* < 0.5 versus model group.

According to the results of adipocyte glucose depletion, phillyrin was selected to investigate the mechanism of improving IR. The effect of phillyrin on the expression of proteins related to PI3K/Akt insulin signaling pathway was investigated. The results showed that phillyrin could significantly increase the protein expression of PI-3K, p-IRS-1, p-Akt, and GLUT4 protein compared with the model group (Figure 3). And phillyrin could significantly promote p-Akt and p-IRS-1 and increase the expression of PI-3K and GLUT4 protein at the concentration of 200 *μ*M compared with other concentrations.

## 4. Discussion

As a Traditional Chinese Medicine,* F. suspensa* is the main raw material for Yinqiao detoxification granule, Shuanghuanglian oral liquid, Qingrejiedu oral liquid, Shuanghuanglian powder injection, and Liancao detoxification oral liquid [28, 29]. Previous researches indicated that* F. suspensa* had an improvement effect on diabetic complications such as hypertriglyceridemia [30, 31]. It is speculated that* F. suspensa* is closely related to the treatment of diabetes, and there is no research on* F. suspensa* improving insulin resistance and its potential mechanism. Therefore, the pharmacological effects of* F. suspensa* on hypoglycemia were investigated in this paper.

Adipocytes are one of the main target cells of insulin, which not only play a crucial role in energy balance, but also act as cytokines secreted by endocrine organs and regulating systemic metabolism [7]. Therefore, this cell line is one of the commonly used cell lines for the study of insulin resistance* in vitro*, and adipose tissue has been recognized as a promising therapeutic target for drug development [32]. Preadipocyte 3T3-L1 is selected to explore the pharmacological activity of* F. suspensa* in improving insulin resistance. Dexamethasone (Dex), a glucocorticoid, activates the signaling pathway by inhibiting insulin, thus preventing the transfer of GLUT4, resulting in insulin resistance. Dexamethasone is often used as the model drug of insulin resistance at the cellular level [33]. Therefore, dexamethasone is selected as the model drug in our research. Vanadium is one of the essential trace elements in higher animals, and studies have found that vanadium produces insulin-like effects in many cells* in vitro* [34–36]. This is consistent with our research, and sodium orthovanadate is selected as a positive control drug.

**Figure 4:**
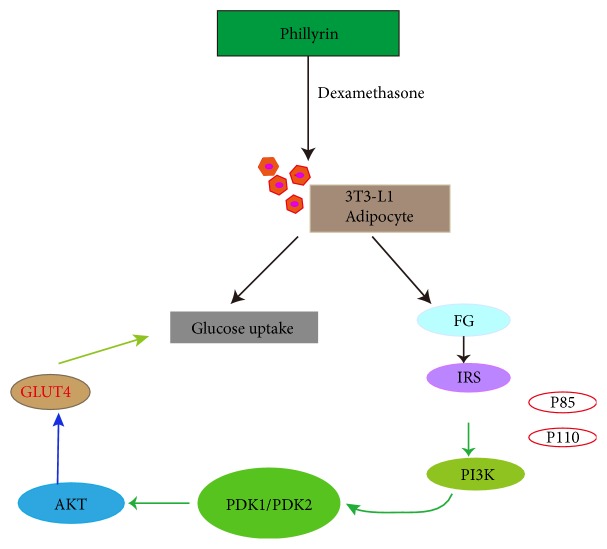
The mechanism of phillyrin to improve the insulin resistance.

PI3K/Akt signaling pathway is one of the pathways of insulin action, which is closely related to the role of IR. Insulin binds to its receptor and its substrate, causing phosphorylation of receptor tyrosine residues and exerting its hypoglycemic effect [37]. In our research, phillyrin could activate the protein of PI3K by increasing the phosphorylation expression of IRS-1 protein (Figure 4) [38, 39]. It has been reported that the absence of IRS-1 or phosphorylated IRS-1 in muscle tissue and adipose tissue is associated with a high risk of diabetes mellitus [40]. Akt is a downstream signaling molecule in the PI3K protein pathway and also a core protein in the PI3K pathway [41, 42]. When Akt protein is activated, it can promote the transfer of GLUT4 from the nucleus to the cell membrane, so it plays a role of glucose transfer and reducing blood glucose. The results showed that phillyrin could significantly increase the expression of PI3K, p-IRS-1, and GLUT4 proteins at concentrations of 200 and 100 *μ*M. Phillyrin, isolated from* F. suspensa*, is a kind of lignans which are natural phenolic compounds with excellent biological activities and have been widely used in medicine and food. In recent years, the pharmacological investigations of lignans have been widely concerned. There are many reports about the hypoglycemic effect of plant extracts, flavonoids, carbohydrates, and other natural products. However, there are few reports about lignans [43, 44]. Studies on the improvement of insulin resistance by lignans included aminophenol and phillyrin [45]. Aminophenol, a fat soluble and flaxseed lignan, was studied with mice induced by a high-fat diet. Phillyrin exerts a beneficial effect on adipocyte dysfunctions induced by tumor necrosis factor-*α* (TNF-*α*). It indicated that phillyrin may play a role in improving inflammatory changes and insulin resistance in obese adipose tissue [46]. It is different from the mechanism with our research to improve insulin resistance. Therefore, it is the first time that it was reported that lignin compounds improve insulin resistance by the PI3K/Akt signaling pathway.

In summary, phillyrin in* F. suspensa* could improve insulin resistance through adipocyte glucose uptake and active PI3K/Akt insulin signaling pathway.

## 5. Conclusion

Phillyrin in* F. suspensa* can promote glucose uptake in insulin resistance 3T3-L1 adipocyte. The mechanism may be exerted through activation of PI3K/Akt signaling pathway.

## Data Availability

The data used to support the findings of this study are included within the article.
